# Medico-Legal Jurisprudence.—Important Trial

**Published:** 1855-04-01

**Authors:** 


### MEDICO-LEGAL JURISPRUDENCE.—IMPORTANT TRIAL.

### HIGH COURT OF JUSTICIARY, EDINBURGH.

### WILFUL FIRE-RAISING.

THIS Court met for the trial of Dr. George Lillie Smith and Robert Campbell, for the crime of wilful fire-raising, at Haughs of Kinnaird, near Brechin.

The Lord Justice-Clerk, Lords Cowan and Deas, were on the bench. The Lord Advocate and Mr. Donald Mackenzie conducted the prosecution; the Dean of Faculty, Mr. David Mure, and Mr. John Millar, appeared for Dr. Smith; and Mr. G. Patton and Mr. A. B. Shand for Campbell.

The prisoners were charged with having, on the 30th of September, or the 1st October, in or near the stackyard of the farm of Haughs of Kinnaird, parish of Farnell, Forfarshire, occupied by Mr. John Smith, wilfully set fire to one or more stacks of grain, by applying to them lighted matches or other ignited substance—the fire thus wilfully applied having taken effect, and burned the whole stackyard, containing altogether fifty-five stacks of grain.

The prisoner Smith is a man of florid aspect, and of middle age. The prisoner Campbell is an old man of simple appearance.

The pannels pleaded not guilty, and the case went to trial. A special defence was put in for Smith, that at the time of the fire he was insane; and for Campbell, that Smith was liable to get excited, and that his conduct under that excitement was calculated to alarm and overawe those with whom he came in contact.

Mr. John Smith, farmer, Haughs of Kinnaird, deponed—My wife lived apart from me for some time, but returned home in the beginning of September last. On the night of the 30th September, at eight o'clock, I was in my stackyard, when all was right. The household went to bed between nine and ten. I was awakened a little after one o'clock, and found the stackyard on fire. I found a number of the servants had already collected. I endeavoured to stop the fire, but did not succeed till all the stacks were burnt. There were fifty-one stacks and four small huts destroyed. I sent to Brechin and to Montrose for fire-engines. Brechin is about four miles distance. The steading is about two miles from the Bridge of Dun, and six from Montrose. Two engines came, and assisted in putting out the flames. There was about half a stack saved, but even it was much scorched and damaged. I estimated the value of the stack-yard at that time at about 2000*l*. It would now have been worth considerably more, from the rise in prices. When I got up there was a slight wind blowing from the north-west. I think its direction became changed afterwards. The farm-steading stands between the house and the stackyard. There is only about three yards space between the steading and the stacks. The house is at the other side of the steading. I suspected immediately that Dr. Smith had set the stackyard on fire. He had sent a threatening letter to my wife some days previous.

The following letter was then read by the clerk of Court:—

"Monday, September 25.

"MY DEAR MRS. SMITH,—I still call you so for all the iniquity you have done me.

"I leave it to yourself to think. (I'll keep all my promises to you. I'll do nothing without telling you.)

"As to your return to Mr. Smith, I should have been as happy to give you my arm to go back as I was to lead you away had you told me. You have much to think of.

"All I ask of you is an interview with me, or write me. My intentions at present are determined thereby. You know my proposed movements, but they shall be delayed according to circumstances.

"I have been ready to help you in trouble, but be assured I am as ready to act when I am opposed. I am not one that will be *shuffled.*

"I ask of you to write me first, and then give me an audience. If you do not do so, I shall have one perhaps less agreeable to you. As your friend, as I have ever been, I ask you to do so. Blame me not, but my blood is boiling, and retribution I shall have in one shape or another. I care not though this comes to his honour's hands—you may show it to him.

"Your happiness *depends on your answer to me.* I am not in trim to be trifled with, or *yet duped.*

"For all you have done against me, and insult offered me, I say

"My dear Mrs. Smith, ever your sincere friend,

"Geo. L. Smith.

"P.S.—Give me an answer, or you shall everlastingly repent it. G. L. S."

On the back was written—

"I confess I walk out of the course of a gentleman in writing this, but it is only my regard for you that has caused me to do so. Recollect, for once and all, I am one *who will act as I say.* G. L. S."

That letter was brought to my wife by a girl named Clark, a daughter of Dr. Smith's housekeeper. Smith had a residence in Montrose, but he had been in Edinburgh most of the time my wife was there. During the greater part of last year I was not on speaking terms with him. I had reason to know, from the report of the person my wife was living with in Edinburgh, that Dr. Smith was displeased with her for returning to me. The policeman came down about two o'clock in the morning, and I told him who I suspected.

Cross-examined by the Dean of Faculty—I and my wife had been living in a state of separation previous to the fire. She had returned to me on the 1st September, after a separation of six months, by agreement. During that period she had been in Edinburgh. I have known Dr. Smith since he came to Montrose, seventeen or eighteen years ago. I have been intimate with him from the autumn of 1852 till we had a difference about the 14th January, 1854. During 1853, Dr. Smith frequently complained of being unwell, and appeared excited. At first I thought it proceeded from illness, and the state of his affairs; but latterly I ascribed it to his taking too much drink. I ascribe it to that cause now, more so than ever.

By Mr. Patton—I had seen Campbell either once or twice before. I never spoke to him. I saw him once in Dr. Smith's house.

By the Court—While in my house Smith had opportunities of getting drink without my knowing it. The sideboard was left open, he having complained of my wife for having locked it. I do not know of my own knowledge that he took drink from the sideboard, but when he slept in my house he got wine placed in his bedroom. He sometimes got into violent passions, and used very violent language. I would not say that these were the effects of drink altogether, but he complained of his being unwell, and I certainly thought he was taking too much drink, and that this was partly the cause of his violence.

By the Dean—He was living a considerable time in my house, from the 4th December, 1853, to about the 16th. He was suffering from ill-health. I insisted on Dr. Booth or some medical man remaining constantly with him till he got better. Before the 4th December, I had had a message from Dr. Smith that he thought he was dying. I went to see him. He pleaded with me to allow him to come to live in my house. I consented to his coming on the condition of his bringing his housekeeper to nurse him. I would not, on any account, permit my wife to do so. He is no relation of mine whatever. He came out to me on Sunday, the 4th, with Dr. Officer, his partner in business, who went away again to Montrose. On the Monday I sent for Dr. Booth, and insisted on his staying with him. On the Friday following he came into the room in his night-shirt, while we were sitting at tea, and in an excited state. It was then that he swallowed several glasses of brandy, which we could not prevent him drinking. His medical man had wanted to confine him to six glasses a day, but he would not be limited to that. I afterwards found he was getting about a bottle and a-half of wine a-day. The next week, on a soothing diet, he got rapidly better, and by Friday following he was going about my stack-yard with Dr. Booth, shooting pigeons. Dr. Smith was married, and had been separated from his wife; and during his excitement he spoke much about that separation. It was in the preceding May he first told us of his separation from his wife, and it was about that time I began to think he was falling into habits of drinking.

Margaret Smith, wife of last witness—I have known the prisoner, Dr. Smith, several years. I had a dispute with my husband about the beginning of last year, and I went to reside in Edinburgh. While there I occasionally called to see Dr. Smith, as a friend. I went back to my husband in September. Dr. Smith did not know I meant to return. After I returned I got a letter from him by a daughter of Mrs. Clark. I gave it to my husband, and sent no answer. I got another letter on the Saturday night, two hours before the fire. I tore it up. Some of the pieces were preserved. These are the fragments of that letter now shown me. All I read was that he expected me to write to him again. I answered neither of the letters.

By the Dean—My acquaintance with Dr. Smith commenced about six and a-half years ago, after I was married. I have never noticed any change in his manners; he has always been the same ever since I knew him. He was allowed brandy, and generally drank his allowance, which was two glasses a-day, I think. When I saw him in Edinburgh, he complained of ill-health. He was at times excited, at other times not so. So long as I knew him, particularly for three and a-half years past, he was subject to fits of excitement.

By the Lord Advocate—I know Dr. Smith intended to go abroad while I was living in Edinburgh. He said he intended to apply to Government for an appointment. I did not know when he got one.

Alexander Officer—I have been partner with Dr. Smith in Montrose since July, 1853. I have known him for six years. Dr. Smith lived some time at Laverock Bank, Trinity. He had gone to London and returned to Montrose about eight or nine days before the fire. He expressed disappointment at Mrs. Smith having returned. On the Monday following he wrote a letter to Mrs. Smith, and read it to me. I advised him not to send it, but he did not take my advice. He told me he had been at the Haughs before writing the letter, but he did not say what he wanted, or if he had seen any one. He said Smith the farmer had endeavoured to injure him by imputations on his character. He said he wanted two questions answered—that he was determined to have them answered before going away. He used threats of revenge, but he did not specify what mode of revenge he would take. I heard of the fire at the Haughs, and that Mr. Smith had suspicions of Dr. Smith. I went with Mr. Smart to Noranside, and found him there with Miss Carnegy and Dr. Steel. Mr. Smart told him what suspicions were abroad. He asked him if the houses were burnt. Mr. Smart said they were not. He asked if Mr. Smith was burnt. Mr. Smart said he was not. He said it was perhaps as well, as burning was too good a death for him. He did not say whether he had done it. The same afternoon I was alone with Dr. Smith. He told me he had been at the Haughs. It was my impression that he meant the previous night. I advised the others who were there that Dr. Smith was in an unfit state to be at large. I said he would be apprehended on suspicion, even though innocent, and that the effects might be very bad on him. I recommended that, as he was to take a voyage at any rate, he should go away immediately. It was ultimately settled he should go to Dumfries Asylum, or rather that he should go on a visit to Dr. Brown at Dumfries, with the view of being quietly conveyed there. I took Mr. Sommerville's gig to Montrose, where I packed up Dr. Smith's things, and addressed them in the girl Clark's name. He was to meet her at Coupar-Angus station. He was apprehended on the Monday in the Strathmore Arms, Coupar-Angus. Dr. Smith drank considerably. He first spoke of going abroad eighteen months ago. I recollect his receiving a letter from the Colonial Land Office in September, requesting him to present himself for examination before the Commissioners.

By the Dean—I was decidedly of opinion that Dr. Smith was insane. I had been acquainted with him about six years. I have treated him for congestion of the brain and disease of the liver. The latter disease was very severe, and I think it contributed to and aggravated his other complaints. He has been treated for the same complaints by Dr. Steel of Forfar and Dr. Booth. He had laboured under this illness since the spring of 1853. About December, 1853, there was an appearance of paralysis arising from that disease. In September we had a consultation with Dr. Christison, of Edinburgh, about him. We thought there was no hope of his recovery from the complaints under which he suffered. We expected his case to result in insanity. He was frequently in a state of great excitement, but sometimes very dull. The Smiths seemed to occupy all his thoughts. He took Mrs. Smith's part in her differences with her husband. There was nothing in these differences that would have excited any sane man in his position. Dr. Smith was in practice eighteen years in Montrose. For some time he had a great business, and was very much esteemed, and held several public appointments. For the last eighteen months he laboured under an affection of the mucous membrane of the throat and stomach, which caused blood to come out at his mouth. He was restless and suspicious. He said he needed to keep a sharp look-out, as parties were plotting against him. In this state of diseased mind he quarrelled with his wife, and separated from her.

By the Lord-Advocate—I understood Dr. Smith to accuse Mr. Smith of improper intimacy with his wife. I first formed the opinion Dr. Smith was insane in December, 1853. During these fits that came upon him I considered him insane. They arose from congestion of the brain, aggravated by disease of the liver. I don't think these fits arose from drinking, though they were much aggravated by it. I was aware of Dr. Smith having obtained an appointment. I never said to any one he was qualified to hold it. I have seen him tipsy three or four times, but he was not a drunkard.

Charles Sommerville, merchant, Montrose—I have known Dr. Smith since 1838, and intimately for the last ten years. He was generally a sober man, but in company he could indulge freely. I could scarcely say I had formed an opinion of his sanity or insanity, but I have seen him violently excited. At times I thought he was right enough in his mind; at other times quite the reverse. He generally carried a pistol with him, and sometimes a sword. He often spoke about a list of persons he had made out, whom he was to shoot or stab, and he often used threats against them. He put every one in his "list" who offended him in any way.

William Gray, apprentice to Alexander Mill, haircutter, Montrose, identified a thick stick he had sold to Campbell. James Anderson, tacksman at Leuchland toll-bar, deponed that Dr. Smith and Campbell passed the bar in a gig at ten minutes to eleven on the Saturday night; James Wilson, hostler, Commercial Inn, testified to Dr. Smith coming with a horse and gig about eleven o'clock to be put up for the night, and leaving it; and James Fleming, boots at the inn, deponed to Dr. Smith coming into the house at three in the morning with his boots and trousers very dirty, and appearing as if they had been wet.

Miss Carnegy, of Noranside, said—I have known Dr. Smith about seventeen years. I remember seeing him at Noranside on the Wednesday before the fire. He talked of Mrs. Smith having gone back to her husband, and complained that she had done so without consulting him, after she had asked him to be her adviser. I saw him again on the Sunday. He told me that he had burned the stackyard at Haughs of Kinnaird, having fired it, he said, with a lucifer-match. He said he expected to burn the farm-house, but that a change of wind had occurred. He said he had had pistols with him, and that he had hid them near the house. On the Tuesday afterwards I got a letter from Dr. Smith from Coupar-Angus. He said he had had a friend at the Haughs, but that friend deserted him. I afterwards found that that friend was Campbell.

Cross-examined—He spoke a good deal about pistols. He said he had three pairs, and that they would fire twelve shots. I have known Dr. Smith intimately seventeen years, and had great friendship for him. He was a gentleman of good character, and much esteemed in the neighbourhood for his good qualities, and his devotion and benevolence to his patients. About January, 1853, a very marked change came over him, and his illness got gradually worse. He became extremely irascible, and this for causes entirely inadequate. In fact, my apprehension was that he would be some day arrested for an act of violence. He lived with me for some time as a boarder.

By the Court—When Dr. Smith told me that he had fired the stack, his manner was wild, but not more so than I had previously seen it. My impression was, that he did not seem to think he had committed any crime. I believe that, on the Sunday morning, when he came to me, he was not a responsible agent.

Several witnesses were called to prove Dr. Smith's journey to Coupar, and to his apprehension there, and to identify Campbell's stick, which was found floating down the Esk. Isabella Baird deponed to a conversation with Campbell, in which the latter stated that, after leaving the horse and gig, Smith proposed to take a walk, and that the Doctor took out a naked sword, and made Campbell walk before him.

Robert Smart, corn merchant, deponed that on the Monday previous to the fire he had signed a certificate as to Dr. Smith's fitness for an appointment under the Emigration Scheme. In cross-examination, he said that, from the alteration in his manner as evinced after the fire, he would not have given such a certificate, as he then considered him insane. On re-examination, he said Dr. Smith was always of an excitable temper.

John Burness, surgeon in Montrose, had known Smith for four years. He had always been of an excitable temper, but he had never seen any symptom of insanity about him. He indulged somewhat in drink, and perhaps affected his health by it.

Alex. Smith, surgeon in Forfar jail, said that when first brought to prison, on the 2nd October, Dr. Smith was labouring under considerable excitement, caused apparently partly from indulgence in liquor, and partly from the position in which he found himself. That excitement subsided very much the following day, when he was much more composed and quiet. Saw him regularly fully twice a-week, and frequently spoke two hours with him at a time. He spoke of his previous history and temperament in a rational and connected manner. He stated that about eighteen months previously he fell into bad health, caused by family vexations, and was naturally extremely irritable. In all these conversations perceived no aberration of intellect whatever. Saw him again last week. My opinion is, and I have no doubt whatever, that he is a sane man. I think it exceedingly unlikely that if he was insane when first confined I would not have observed some symptom of it during his confinement.

Cross-examined by the Dean—When apprehended, the prisoner's bodily health was in a very unsatisfactory state. I have also some doubts of his brain being in a healthy state; but it is difficult to give a definite opinion on what diseased action may be going on in the brain, so I can't pronounce with any degree of certainty whether his brain is affected or not. His complaints are enlargement of the liver, but curable, I should think; a bloody oozing from the gums, which indicates a general weakness of the system, and arises from an imperfect assimilation of the food; digestion also defective. Don't think there is any diseased action of the brain at all. Of his ailments indigestion would affect the mind very much, and render him irritable; but none of them, I think, are calculated to produce insanity. I ascribe his excitement to indulgence in intoxicating liquors; but if that was wrong as matter of fact, it might arise from indigestion acting sympathetically on the brain. Could find nothing else to account for excitement or extravagance.

Re-examined—Traces of congestion of the brain are very occult, and symptoms supposed to arise from it often are found in reality to proceed from different causes. Dr. Smith's complaint would be aggravated by drinking. Never saw any excitement excepting the first day.

By the Court—When put in prison he was not drunk, but had quite the appearance of a man after a severe debauch a day or two before. Once he alluded slightly to the charge preferred against him, and asked what they would do with him. There was no appearance during his residence in the jail of his wishing to feign insanity.

By the Dean of Faculty—Was afraid of *delirium tremens* at first, and ordered stimulants to be given when necessary. It, however, did not ensue, and he got some stimulants once or twice during his confinement.

Dr. William Malcolm, physician to the Perth Asylum, read the notes he had taken of several visits which he had made to Smith while in prison. He spoke quite rationally in all the conversations he had held with him, and never exhibited any appearance of aberration of intellect. The result of his first examination was, that so far from being insane, he was an acute and clear-headed man. On a subsequent occasion he was highly indignant on hearing that the plea of insanity was to be made for him, and said he would rather be shot than shut up for life in a mad-house, when he was, and had all his life been, perfectly sane. Dr. Malcolm, in his examination by the Lord-Advocate, said he had no reason to change the opinion he formed at first with respect to the prisoner's insanity. Had Dr. Smith been gradually exhibiting symptoms of insanity for eighteen months previous to his imprisonment, he thought it impossible he could have recovered, so as not to have exhibited some sign of it during his confinement. Mental distress, aggravated by strong drink, would naturally produce paroxysms in a man of irritable temper. In answer to the Dean, the witness further stated that he never found a case in which a patient exhibited an insane delusion on one subject more than another. He added, that he found cases in which men whom he knew to be insane were anxious to make themselves out as sane.

C. Dickson, Sheriff-Substitute at Forfar, said that Dr. Smith was brought before him for examination on the 2nd October, and the opinion he formed was that he was then in his sound and sober senses.

By the Court—There was certainly a slight nervous appearance, but it did not seem to him more than what might have been expected in one brought up on so serious a charge.

Alexander Warden, clerk to the Sheriff-Clerk of Forfar, also gave it as his opinion that Dr. Smith's declaration was freely and voluntarily emitted, and when in his sound and sober senses.

The declaration of Dr. Smith, which was simply that he declined to answer any question, and the declaration of Campbell, were then read. Campbell, in his declaration, stated that on Saturday night, the 30th September, Dr. Smith met him on the streets of Montrose, and, after treating him to a beefsteak and some tea; they proceeded in a gig to Brechin, thence to the Haughs of Kinnaird. Dr. Smith used threatening language against Mr. Smith of the Haughs, and said he was going to set fire to his farm-yard, and that he would shoot Smith if he came out. Campbell went on, as he was afraid of his life, for Dr. Smith told him he had a pair of loaded pistols. Dr. Smith then proceeded towards one of the stacks, and in a minute afterwards he saw the stack in flames. Dr. Smith wished him to put a rag dipped in turpentine, which he said he had on his person, in one of the stacks, but he declined to do so. On seeing the flames, he made off, and did not see Dr. Smith after that.

This closed the case for the prosecution, and the court adjourned for a brief interval to enable the Judges to attend in the First Division at the presentation of the letters appointing the new Judge and Solicitor-General.

EVIDENCE FOR THE DEFENCE.

George Smart, merchant, Montrose—who was, on the re-assembling of the court, called and examined by Mr. Muir—said Dr. Smith had at one time enjoyed considerable practice in Montrose, and occupied several public situations with great credit. Remember Dr. Smith's accompanying a Colonel Frazer to London, with a view to the latter being put in a lunatic asylum. He died in London, and when I next saw him he seemed very much affected by the Colonel's death. From that time I have observed a great change in Dr. Smith. He was a man, generally speaking, of sober habits; but ever since he has been highly irritable. He was in bad health in 1853, when Dr. Christison attended him. He frequently told me his disease lay at the back of his head, that his mind was affected, and if he could cure his mind, his body would be cured in forty-eight hours. At that time I observed great excitement in his manner. He threatened to take the lives of several parties who he supposed had injured him; but these complaints, I believe, were entirely imaginary. He asked me to carry challenges to one or two, but I reasoned him out of it. He has also conducted himself in a reckless and extravagant manner. I have seen him throw down swords and guns on the table—threatening to run parties through the body, and cut them open. Immediately afterwards I have seen him spit mouthfuls of blood. The attacks seemed to come on suddenly, and I believed him to be perfectly sober. He used to come to my house at all hours, and when he got a bed I believe he could not sleep. He often complained of violent pains in his head and chest, which induced me one night to put him into a bath. He said next morning if I had not done so he was satisfied he would have been dead in fifteen minutes. On one occasion, when he was dining with me, he went to the garden and lay down, saying, in reply to my entreaties to get up, that he had often lain all night in his garden, and next morning found his hair frozen to the grass. This was in 1853. He went in the end of that year to the Haughs, where I saw him twice, weak and ill in bed. In Edinburgh I saw him in August, 1854, in a hotel, where he became most violent and excited in his manner about the way in which the fish were boiled. He has a wife and family. For some time Mrs. Smith has been residing with her father in Liverpool. There is not the slightest foundation for an insinuation that I was too familiar with Mrs. Smith; there is not a more virtuous woman in the country.

By the Court—Dr. Smith appeared generally suspicious, and I think the separation from Mrs. Smith was owing to some causes which he exaggerated to himself. Their five children were left behind when Mrs. Smith went away, but he always behaved very kindly to them. He imagined the public were set against him, and therefore he could not come near his own house. At that time and from all these circumstances I considered his reason quite overthrown, and thought he should be sent to an asylum—an opinion which was shared in by Dr. Booth and others in 1853. He was very much respected as a medical man, and great forbearance was shown to him. He proposed at one time to take a partner in his profession—a proposal which, in the unsettled state of his mind, I cordially seconded.

William Jameson, merchant, and formerly Provost of Montrose—I know Dr. Smith, who was professional adviser to my family for eight or ten years. Both in his private practice and public situations he was very highly esteemed as well for his professional acquirements as his private character. Within the last two years I have observed a very considerable change in him—particularly in his conversation, manner, and dress. At first he was a gentlemanly man of refined feelings, great delicacy in his conversation, clean and neat in his dress. Within the last two years all these were changed: coarse in conversation and slovenly in his dress to a disgraceful degree; but I never saw him otherwise than sober. His extravagances in Montrose were frequent. He threatened to shoot Mr. Boyd, the banker, and wanted Dr. Booth to carry a challenge to him. He had an idea Mr. Boyd had interfered with the Inspector of Factories to have his appointment of surgeon of factories given to another.

Peter Matthews, guard on the Scottish Central Railway, stated, with reference to the collision on that line in April, 1854, that Dr. Smith was one of the passengers in the centre compartment of a carriage. After the accident he appeared almost insensible. Complained afterwards of the shock. Saw him afterwards sitting down at the bottom of the embankment, but he would not rejoin the train, though he afterwards did. He appeared greatly hurt by the concussion.

James Wilkie, the other guard, gave similar evidence.

James M'Gregor, of the National Hotel, said Dr. Smith lived in his house in the latter part of August last. Occasionally noticed his conduct was remarkable—sometimes very excited, sometimes very melancholy, and at others very merry. Heard the gentlemen in the public room often say that he should not be left alone. He carried a dirk-like knife about with him. This he frequently exhibited in the public room. He used to lay it down beside him when he was taking his food. He said he got it from his father, who desired him to take care of it. "It had done deeds before, and it might do so again." The exhibition of the knife was made matter of complaint, and witness took the knife under his own charge. After that, saw him with a large carving-knife in his outside pocket as he was going out at the door, but took it from him. He said he was constantly hunted by spies looking after him, who stopped when he stopped, and turned round after him. He added, he had warned them against such conduct, and he took the knife for protection when he went out. There was some person, he said, he wanted revenge on, but he did not mention the cause of it. He asked witness if he would go out and second him in a duel. He refused, but promised, in order to pacify him for the time, to procure another second. He mentioned some one had injured him in his family—that some one had attempted to seduce his wife. He was sometimes in a state of great excitement in the public room. Nobody came into the room but he invited them to partake of what he was having. He left at ten o'clock on Wednesday morning. He came with a person in the evening—left immediately, and came back about half-past one. He wanted him to go and get his plaid, and proposed they should go and sleep all night in it on the Calton Hill. Witness remonstrated, on which he refused to come in, and said he would stand at the door all night. He then shut the door, on which the prisoner rung the bell violently, said he was insulted, and would leave immediately. Assisted to pack his luggage; he gave him his knife, and as he was in a state of great excitement against him, he left him and a waiter in the room. He afterwards heard a great noise, and heard him declare he would have the heart out of him. He next heard him rub the knife against the steps on the stair, so his apprehensions being serious he locked himself in. He did not drink much while he was staying in the house. He could take his glass, but he never saw him the worse of liquor.

By the Lord-Advocate—I have no notion of how much he would take in a day; he could always walk and talk. He came back after the last affair and made an apology to me. He said he was in one of those fits to which he was liable. He was then perfectly quiet.

Thomas M'Lean, waiter in the National Hotel, corroborated the last witness.

Dr. Christison—I was sent for in 1853 to visit Dr. Smith. I became first acquainted with him as a student and pupil in the University, afterwards in practice. I always regarded him as a very intelligent practitioner. I found him at Laurenceside, and visited him with Dr. Steel and Dr. Officer. The result of my observations was, that he laboured under a great enlargement and disease of the liver, great disturbance of the circulation, very rapid pulse, 120 to 130. He also laboured under considerable mental excitement. I ascribed it to the explanation given to me at the time, that there was a real cause—a great disturbance in his domestic circle. There was a probability of the cause being adjusted. He also complained at that time of an uneasy sensation in the back of the head, and heat generally in the head—want of sleep. His health was such that I formed a very clear opinion on two points, that he was very seriously ill, and also that there was great danger of his passing into a state of insanity. There were symptoms of an affection of the head forming, and there was a risk of his being constantly exposed to serious sources of excitement. The other medical men agreed with me in opinion. In January, 1854, I entertained hopes of recovery, but still the symptoms were serious. I again saw him last Sunday in the Calton Jail. I found him very poor in health. I found a large tumour in the upper part of the abdomen, evidently connected with the liver. His expression was so much that of one who had been recently intemperate, that I asked the governor if he had had spirits, and he said no. I then ascribed it to physical disease. I have not seen him at any time in a state of insanity. I would think it very probable he may occasionally be in a state of insanity, notwithstanding the calm and quiet state in which I found him in prison. A medical gentleman sent to see him, and merely sitting and talking with him, would not, except perhaps by accident, discover any trace of insanity. The form of insanity I would expect in his case is unreasonable suspicion, and strong feeling of resentment on account of imaginary injuries; but, of course, any form of insanity might arise—though the one I have mentioned is the usual form. A man under the influence of such delusions I would pronounce insane for the time.

By the Lord-Advocate—When I saw Dr. Smith I did not see any evidence of insanity, but as to the question whether I believed him insane, that is a different question. Several of his statements I considered to be delusions, and his general mode of statement indicated insanity. This was on Sunday last; but on no former occasion did his conversations show him to be insane. From anything I saw he was then able to distinguish between right and wrong. My apprehensions as to his sanity arose from the symptoms of cerebral disease. I cannot pronounce with certainty, but I labour under a very strong belief that cerebral disease has existed for some time, insomuch, that were he a patient of mine, I would treat him for cerebral congestion or some more permanent organic disease. I infer that from the great restlessness at night, height of pulse, heat in the head, an attack in Forfar Jail, where he seems to have lost recollection and fallen to the floor, from his having at various periods had imperfect paralysis in one of his limbs, these, combined with his general state and appearance of his eyes, are so strong indications of the state of the brain that I would treat him for congestion. Congestion, however, is undoubtedly not only consistent with sanity, but with the most perfect bodily health. It is the character of persons labouring under this insanity to show it very readily to persons beneath them in station, or with whom they are very familiar; but to keep it out of sight of those who are their equals or above them in station, or not familiar with them. As to whether the surgeon of the jail or myself could form the best opinion, I think the surgeon of the jail would have a very good opportunity of forming a correct opinion. Imprisonment on a criminal charge is undoubtedly a subject of mental disturbance. Perhaps, however, confinement in a jail would have the same effect as removal to an asylum, in removing causes of excitement.

By the Court—It would not have surprised me, from what I know of the history of cases of the kind, to find such a patient labouring under strong delusions on Saturday night, and comparatively quiet on Monday or Tuesday. After such an outbreak it is not uncommon that there is a reaction and depression in the physical system.

Donald M'Kay, governor of Forfar prison, stated that from the 2nd October to the 11th inst., the period during which Dr. Smith had been confined, he had only on three or four occasions had a glass of punch, and was only induced to take it after some persuasion. He never asked for it except once, after eating something that disagreed with him. He exhibited no desire for it at all. He varied much, particularly during the first ten or twelve days. He was sometmes quiet and sometimes excited. He was then very bad. He was never very frightened for him; but sometimes he would rather have been out of the cell than beside him. He had seen him in such a state that he could imagine nothing too mad-like for him to do. He was at first impressed, from the appearance of the eye, that Dr. Smith was insane, and the impression was confirmed by subsequent events in his conduct.

Dr. Brown, medical superintendent of the Crichton Asylum, Dumfries, considered Dr. Smith, when he knew him at Montrose, to have been a most intelligent practitioner, and a respectable member of society. Their acquaintance was renewed in 1854, when he asked his opinion relative to an attack of paralysis, but his letters were not those of Dr. Smith of former years. In the first place, they were not those of a man of education—sometimes not intelligible, and contained great suspicions, conspiracics, imaginary injuries, and generally marks of great excitement. His impression was that he was bordering on some form of mental disease. Visited him in December last, and found him so changed in the expression of the eye and in the features generally, that he should not have been able to recognise him. From the extreme rapidity of pulse—alteration in the mode and precision of articulation—pain and uneasiness on the skull being slightly struck—and the generally diseased condition of the body—sleeplessness, &c., he inferred disease of the brain. He concluded there was a change in the structure of the brain, and, certainly, if he had been put under his charge, he would have treated him for that affection. The prisoner spoke of the criminal charge against him at one time as a practical joke—then as a righteous judgment. He also mentioned quite seriously that he had a positive power under the head or bed of Mrs. Smith of Kinnaird, to blow her up. This he (Dr. Brown) treated as a delusion. He vowed vengeance also against some people whom he styled enemies. He regarded all these as manifestations of the disease of the brain, which he before had inferred. Believed him at that time to be quite insane, quite incapable of distinguishing between right and wrong, and not responsible for his actions. But that applied only to certain times; because there were occasions when he was perfectly calm.

William Steel, surgeon in Forfar, corroborated the opinions of Dr. Christison and Dr. Brown as to the insanity of the prisoner, and the disease under which he was labouring.

Thomas Morrison, superintendent of the Notting Lunatic Asylum, and formerly of the Montrose Asylum, was examined to the same effect.

Dr. Alexander stated that he had become acquainted with Mrs. Smith, of Montrose, in 1854, who got lodgings in Edinburgh through his recommendation. Afterwards had occasion to visit her professionally. Found the prisoner came to Edinburgh, and met him, on the part of Mrs. Smith, at Laverock Bank, and on other occasions, on one of which prisoner came up to him, and said if he did not mind his own business he would knock his brains out. The threat was again repeated one day in George-street; and he had never given any cause of offence, excepting in preventing his meeting with Mrs. Smith. Never had any conflict with him. Never fought a duel.

This closed the case for Dr. Smith, who was then removed, and the diet continued against him till this morning at nine o'clock.

Catherine Burn—examined by Mr. Patton for the pannel Campbell—stated that she resides near the beach at Montrose. Has known Campbell for twenty years, and ever regarded him as a quiet inoffensive person, unlikely to do any injury to anybody. He attended to the boat of Dr. Smith. The latter came to her house on the 26th of September, and remained for three quarters of an hour, till the tide was high enough to float the boat. He asked for a sheet of paper, and wrote a few lines, giving him the boat, if he should not return to Montrose, on condition of its never being sold.

James Orkney, examined by Mr. Shand—Am a mariner in Montrose. Have known Campbell for many years. He left his house the worse for liquor to go home the night before the fire at Kinnaird Haughs. Never saw him except on that occasion affected by liquor. He is highly esteemed in Montrose.

Provost Mackie, of Montrose, also bore testimony to the good character Campbell had long borne in Montrose.

This concluded the case for the defence, and the Court adjourned at a quarter before six o'clock till next morning.

The Court met again on Wednesday, when the Lord-Advocate addressed the jury for the prosecution. After fully describing the nature of the case, he referred to the trial of James Gibson before the Lord Justice-Clerk, in which his lordship, following the doctrine laid down by the Judges of England in 1843, in answer to certain questions put by the House of Lords, directed the jury that a party, not otherwise insane, convicted of crime committed under the influence of an insane delusion, and for the purpose of redressing or revenging some supposed grievance or injury, or producing some public benefit, was nevertheless punishable according to the nature of the crime committed. In this case the Lord Justice-Clerk also laid down the law that it was not sufficient that the pannel should raise the defence of insanity; he must establish beyond doubt such insanity as to exempt him from punishment by evidence which brought complete conviction to the minds of the jury, who were to decide the question of insanity, and not the medical witnesses who might be called. The Lord-Advocate also quoted from Baron Hume to show that the disorder must amount to an absolute alienation of reason. His lordship then went over the whole case in detail, contending that the circumstances only gave evidence of unbridled passions, excitability of disposition, and irritability of temper, aggravated upon occasions by intemperance. His lordship concluded by saying that, in his opinion, the evidence did not come up to what the law required when a plea of insanity was tendered.

The Dean of Faculty addressed the jury for the prisoner Smith, commenting at some length on the evidence, and contending that better evidence on a plea of insanity existing at the time of the perpetration of the offence, had never been presented to a jury. No doubt, he said, insanity was but a miserable plea to urge. He could not ask them by their verdict to restore Dr. Smith to his former position in society. By the visitation of God he had been stricken with a malady which was the most grievous of all; but though they could not restore him to his former state, they had nevertheless a most important duty to perform. They could do justice in this case. They could affirm, and he apprehended they were bound to do it, that when he committed the offence he was absolutely bereft of reason.

Mr. Patton having pressed the Lord-Advocate privately to withdraw the charge against the pannel Campbell, but without success, proceeded to address the jury, and especially to vindicate him from an incidental remark of the learned Dean, in which he described him as "a tipsy old man," whereas he was of a most respectable character, and of exceedingly sober habits. He then stated that he was prepared to go into the case if the Court and the jury thought it necessary.

The Lord Justice-Clerk said, Counsel must judge of that; but the jury, after a moment's consideration, stated by their foreman that they did not consider it necessary.

The Lord Justice-Clerk, in his charge, said, that while it was right for those who administered the law to attend to the principles of jurisprudence by which the question of punishment was to be regulated, or the liability to punishment determined, these were not considerations for the jury. Their duty was to say, upon the facts, whether the insanity of the prisoner was established; they had to apply the law as laid down by the Court on that part of the case. They must not be too much affected by the gradual declension of a person of talent and respectability into the state in which he was found at last, if they were not satisfied that that was the result of insanity. They must distinctly remember that it lay with the prisoner to prove fully and satisfactorily that he was not liable to punishment in respect that when he committed the crime he was bereft of reason. If that defence were not found tenable, the Crown was entitled to their verdict. Common sense justified the rule of law, which required the prisoner to prove this plea, and they were only acting properly and fairly in requiring that it should be clearly made out. He (the Lord Justice-Clerk) did not intend to enter into the law of the case so fully as he had done in that of Gibson in 1844, as referred to by the Lord-Advocate. To the charge he then gave, he adhered in all respects; and he was glad that that charge had been taken by the best English writers on medical jurisprudence as an exposition of the law on the subject. In the first place, they must understand that the law did not for one instant countenance the notion of moral insanity—that was to say, what was called irresistible impulse, by which a man was driven into crime, while it was not proved that his reason was destroyed. That perversion of moral feeling was not insanity. The view of such cases taken by the law was the doctrine of the Bible—that if a man gave way to temptations, which were strong only because he had long indulged in evil thoughts and angry passions, he was not tempted above what he was able to bear; and, unless there was an absolute aberration of reason, the law held that he could resist, and must resist, promptings to commit an act contrary to law. Then, again, there was no such thing admitted in law as partial insanity, call it monomania or anything else. As was well said by Lord Brougham, if the mind were unsound in one point it was unsound in every respect so long as that which caused the unsoundness existed in the mind. It was not necessary, however, that insanity should be continually and constantly manifested, for a man might be insane at particular times—at one time a fit object of punishment, and at another an unfit object of punishment. The ordinary instances of this were to be found in what were termed "lucid intervals." But the jury must understand that absolute alienation of reason must be proved, a principle well explained by Baron Hume, and not less important in the present case because that profound thinker and excellent lawyer connected it with delusions. He had to remind them that the jury in such a case as this were far better judges of what insanity was than either medical men or lawyers. While he had told them that the pannel must be bereft of reason to be exempted from punishment, they were not to suppose that that implied a state of demoniacal fury. Another consideration was, whether the insanity was heard of for the first time after the commission of the crime, or whether weakness of intellect had been going on progressively, with physical disease, for a length of time, and resulted gradually in alienation of reason. With regard to the prisoner Campbell, if they should be satisfied that the act was committed by Dr. Smith, under the influence of insanity, he (the Lord Justice-Clerk) did not think they would be inclined to bring in Campbell guilty of the act without Dr. Smith. The evidence against Campbell was as bare as it could well be, and it would have been infinitely better had he been produced as a witness, as he might have told what Dr. Smith did on that night. His lordship then proceeded to review the evidence, and his comments upon the leading facts of the case were favourable to the special defence set up for Dr. Smith. His address lasted, on the whole, above four hours.

The jury retired at a quarter past five o'clock, and, after ten minutes' absence, returned the following verdict:—

"The jury unanimously find that the pannel, George Lillie Smith, committed the act of fire-raising mentioned in the libel, but that he was insane at the time of doing so; and find the pannel, Robert Campbell, not guilty."

The verdict having been recorded, Lord Cowan discharged the jury, and expressed his regret that they had been so long detained.

The prisoner Campbell was then dismissed from the bar, and Dr. Smith was ordered to be brought up next day for judgment.

The Court, which had been much crowded throughout the whole proceedings, adjourned at half-past five o'clock.

The Court met next morning at half-past ten o'clock, when Dr. Smith was brought up for sentence.

The Lord-Advocate, in moving for judgment, called the attention of the Court to the case of John Smith, convicted for murder at the Jedburgh Circuit Court, and to that of Isabella Boyd, convicted for the same crime before the Perth Circuit, in both of which cases the pannels having been found insane, the Court adjudged them to be confined for life, or until the further orders of the Court.

The Lord Justice-Clerk thought these cases exceptional, inasmuch as the sentences had been pronounced on Circuit, and not at the High Court, which had a form of its own. His Lordship then pronounced the sentence of the Court, which was as follows:—"In respect of the verdict of the jury, find that the pannel is not a proper object of punishment, and, therefore assoil him *simpliciter;* but, in respect of the insanity found proven, decern and adjudge him to be carried back to the prison of Edinburgh, and from thence to be retransmitted to the prison of Forfar, therein to be confined, subject to the future orders of this Court."

Before the prisoner was removed, the Lord Justice-Clerk expressed some doubts as to how far the prison Board had the power of interfering with him in regard to his place of detention, and his Lordship intimated his opinion that, to obtain the greatest chance of a cure for the pannel, he should be placed in the lunatic ward of Perth Penitentiary.

Dr. Smith was then removed from the bar. He betrayed no appearance of emotion on leaving the dock, and seemed quite indifferent to the prospect of prolonged imprisonment which awaited him.

Counsel for the Crown—The Lord-Advocate, and Donald Mackenzie, Esq., Advocate-Depute. Agent—Mr. J. C. Brodie, W.S.

Counsel for Dr. Smith—The Dean of Faculty, David Mure, Esq., and John Miller, Esq. Agents—Messrs Hope, Oliphant, and Mackay, W.S., and Mr. William Shiress, writer, Brechin.

Counsel for Robert Campbell—George Patton, Esq., and A. B. Shand, Esq. Agents—Mr. David Crawford, S.S.C., and Messrs. Thomson and Savage, writers, Montrose.

